# Use of convalescent plasma in COVID-19 treatment: is clinical severity more important than the intervention?

**DOI:** 10.31744/einstein_journal/2024AO0563

**Published:** 2024-11-26

**Authors:** Valéria de Freitas Dutra, Heitor Duarte de Andrade, Viviane Regina Hernandez Nunes, Gabriella Mafra Elia, Juliane Roberta Dias Torres, Carolina Bonet Bub, Ana Paula Hitomi Yokoyama, José Mauro Kutner

**Affiliations:** 1 Hospital Israelita Albert Einstein São Paulo SP Brazil Hospital Israelita Albert Einstein, São Paulo, SP, Brazil.

**Keywords:** COVID-19 serotherapy, Hospitalization, Inflammation, Dialysis, Immunization, passive, Plasma

## Abstract

**Objective:**

This study compared the outcomes of two cohorts of patients with coronavirus disease 2019 (COVID-19) who received COVID-19 convalescent plasma transfusions between 2020 and 2021.

**Methods:**

This retrospective study was conducted at a tertiary hospital in São Paulo, Brazil. We included a retrospective cohort of patients who received convalescent compassionate plasma, and another group of patients from a previous clinical study. We collected clinical and laboratory data on the day of and 5 days after transfusion. Patients with hematological or immunological conditions were excluded. Statistical significance was set at p<0.05.

**Results:**

COVID-19 convalescent plasma did not affect the outcomes of patients with severe COVID-19 when comparing the two cohorts transfused with different volumes and titers of neutralizing antibodies. Despite improvements in some laboratory parameters, no effect on clinical outcomes was observed. Dialysis negatively affected the length of intensive care unit stay, hospitalization, and mechanical ventilation use. Each higher point on the day 0 World Health Organization scale reduced the probability of hospital and intensive care unit discharge and the risk of mechanical ventilation discontinuation.

**Conclusion:**

Dialysis and the assessed clinical severity represented by the World Health Organization scale on day 0 influenced the outcomes, whereas COVID-19 convalescent plasma transfusion did not.

## In Brief

Dutra et al. studied two cohorts of COVID-19 patients who received COVID-19 convalescent plasma transfusions between 2020 and 2021. They showed that convalescent plasma transfusion did not affect clinical outcomes despite improving some laboratory parameters. Dialysis negatively affected the duration of intensive care unit stay, hospitalization, and mechanical ventilation use.

## Highlights

■ Convalescent plasma did not affect the outcomes.
■ Convalescent plasma improved some laboratory parameters but had no impact on clinical outcomes.
■ Dialysis and the World Health Organization severity score on day 0 influenced the outcomes.

## Graphical abstract

**Figure:**
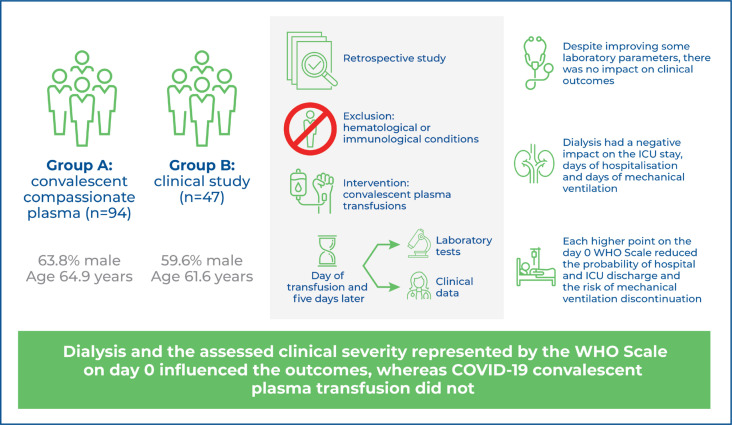

## INTRODUCTION

Since its emergence in 2019, severe acute respiratory syndrome coronavirus 2 (SARS-CoV-2) has become a global health concern. Passive immunotherapy with antibodies has been a treatment option for emerging diseases, and blood or plasma obtained from convalescent individuals has been used to treat infectious diseases for over a century.^(1)^ Coronavirus disease 2019 (COVID-19) convalescent plasma (CCP) has been used in the early stages of the pandemic,^(2)^ especially in clinical trials. Some patients who were not included in the research protocols received compassionate plasma therapy, that is, the use of medicinal products not authorized but in the development process which are offered to patients for whom no other satisfactory treatments are available.^(3)^

In December 2021, the World Health Organization (WHO) guidelines recommended the use of CCP.^(4)^ In 2022, the guidelines from the Association for the Advancement of Blood and Biotherapies recommended that CCP would be more beneficial when higher levels of neutralizing antibodies are used and transfusion is performed early after infection.^(5)^ While most studies have indicated that convalescent plasma confers no survival benefit when used in critically ill patients with COVID-19, the same trials have identified clinical benefits after transfusion, such as viral clearance and reduced supplemental oxygen levels.^(6)^ Although the possible reasons for the conflicting outcomes have been recently reviewed,^(7)^ which factors can impact the results remains unclear, mainly because of the lack of therapeutic alternatives for severe patients in the pandemic and the possible inefficacy of CCP passive antibodies when an inflammatory response has already been established.^(7)^

Studies comparing laboratory test results have shown improvements after CCP transfusion.^(2)^ Blood counts have an important prognostic value for managing patients with COVID-19.^(8)^ Moreover, derived indices, such as the neutrophil-to-lymphocyte ratio (NLR), showed good predictive value for disease severity and mortality.^(9,10)^ In addition, the C-reactive protein (CRP)-to-lymphocyte ratio was found to predict the occurrence of pneumonia in patients with COVID-19.^(9)^ These indices may be used as markers of the inflammatory state of patients during transfusion.

## OBJECTIVE

This study aimed to compare two cohorts of patients with COVID-19 who received convalescent plasma transfusions in 2020 and 2021. To determine the factors that could influence the outcomes, we collected clinical and laboratory data on the day of and 5 days after transfusion.

## METHODS

### Subjects

We included a retrospective cohort of patients who received CCP (Group A, n=94, from January to May 2021) and another group of patients from a clinical study^(11)^ (Group B, n=47, from March to October 2020). COVID-19 diagnosis was confirmed by detecting SARS-CoV-2 RNA using real-time (RT)-PCR. All patients were aged >18 years. The exclusion criteria were previous reactions to blood components, pregnancy, presence of hematological or immunological conditions (including the use of immunosuppressors), and less than 5 days of hospitalization.

In Group A, the criteria for receiving convalescent plasma were <5 days of admission, severe clinical presentation, or moderate disease with the possibility of rapid deterioration. All patients in this group signed an informed consent form to receive plasma and an electronic form for inclusion in this study (REDCap). In Group B, the criterion was severe pneumonia. For all patients, we determined the WHO clinical progression scale^(12)^ score on the day of and 5 days after transfusion.

### Convalescent plasma

Convalescent plasma products were obtained from whole blood or plasmapheresis donations. Donor screening was based on the regular Brazilian criteria for blood donation and negative RT-PCR results from blood and naso-oropharyngeal swabs collected on the donation day. Serum was collected from each donor for the determination of SARS-CoV-2-neutralizing antibodies (nAbs) for further analysis. The nAbs were titrated using a cytopathic effect-based virus neutralization test (CPE-based VNT) against SARS-CoV-2 (GenBank MT126808.1).^(13)^ We calculated geometric mean titers for patients receiving multiple transfusions. All the enrolled patients were transfused with ABO-compatible CCP. Six transfusion reactions were observed and investigated. However, none were severe.

### Laboratory tests

Laboratory tests were performed on the day of and 5 days after transfusion. Creatinine and CRP levels and blood counts were measured. We also calculated the following ratios: (NLR, lymphocyte-to-C-reactive protein ratio (LCRPR), platelet-to-lymphocyte ratio (PLR), neutrophil-to-monocyte ratio (NMR), and lymphocyte-to-monocyte ratio (LMR).

All blood tests were performed at the hospital's clinical laboratory. A complete blood cell count was performed using an automated hematology analyzer (XN-1000-Hematology-Analyzer; Sysmex, Hyogo, Japan) with manual adjustment when indicated. Creatinine levels were measured using a kinetic colorimetric reaction (Jaffe for Roche Cobas) and CRP levels were determined using an immunoturbidimetric assay (Cobas CRP4 C501; Roche Diagnostics, CA, USA).

### Data analysis

Quantitative variables are reported as the mean (standard deviation [SD]) and/or median (interquartile range [IQR]) and were analyzed using *t*-test and Mann-Whitney test, respectively. Qualitative variables are reported as the percentage or frequency distribution and were compared using the χ^2^ or Fisher exact test. To evaluating the difference between days 5 and 0, we used the formula Δ= (D5-D0)/D0. The Spearman's rank test was used to identify correlations. The Cox Model was used to examine the relationship between survival and the quantifying variables.

Data were organized in Microsoft Excel 365 and analyzed using the SPSS software (Chicago, IL, USA). Statistical significance was set at p<0·05.

### Ethics

This study was approved by the Ethics Committee of *Hospital Israelita Albert Einstein*, CAAE: 57841922.2.0000.0071; # 5.707.823.

## RESULTS

This retrospective study included 141 patients with COVID-19 who received convalescent plasma transfusions. Groups A and B were significantly different when considering the intervention: days after symptom onset (8.5±4.3 *versus* 11.09±3.8, p<0.001), time from admission to transfusion (1.5±1.2 *versus* 3.4±2.4 days, p<0.001), volume (242±67 *versus* 491±158mL, p<0.001), and quantity of nAbs [160 (80-2560) *versus* 320 (20-1810), p=0.006]. Table 1 presents the demographic and clinical characteristics of the patients.

**Table 1:** Demographic and clinical data comparing the two cohorts

Variable		Group A (n=94)	Group B (n=47)	Total (n=141)	p value
Demographics					
Age /years#		64.9 (SD: 15.7)	61.6 (SD:17.05)	63.8 (SD: 16.22)	0.249
Gender, n (%)					
	Female	34 (36.2)	19 (40.4)	53 (37.6)	0.623
	Male	60 (63.8)	28 (59.6)	88 (62.4)	
Comorbidities					
	BMI (kg/m^2^)#	28.55 (SD:4.84)	29.57 (SD:4.66)	28.89 (SD:4.79)	0.239
	Hypertension (Yes/No), n (%)	51 (54.3)	26 (55.3)	77 (54.6)	0.905
	*Diabetes mellitus* (Yes/No), n (%)	35 (37.2)	15 (31.9)	50 (35.5)	0.534
Clinical data					
	WHO ordinal scale score on D0	5 (3-8)	6 (4-9)	5 (3-9)	<0.001**
	WHO ordinal scale score on D5	5 (0-9)	5 (0-9)	5 (0-9)	0.958
	ICU on day of the transfusion (Yes/No), n (%)	29 (29.8)	40 (85.10)	69 (48.9)	<0.0001**
	Use of corticosteroids (Yes/No), n (%)*	91 (96.8)	43 (91.5)	134 (95)	0.22

Quantitative variables are reported as the mean (SD) and/or median (IQR) and were analyzed using Mann-Whitney and

# *t*-test. Qualitative variables are presented as the percentage or frequency distribution and were compared using χ^2^ test or

* Fisher exact test;

** p<0.05.

The mean age of the participants was 63.8 years and did not differ significantly between the groups. Most patients were men. The total mortality rate during hospitalization was 12.1% and did not differ significantly between the groups. Most patients (54.6%) had hypertension and some (35.5%) had diabetes, with the mean body mass index (BMI) being 28.89, which indicates overweight. As shown in table 1, the groups differed in severity on day 0, as represented by the WHO ordinal scale and admission to the intensive care unit (ICU) on the day of plasma transfusion.

Blood cell counts and derived indexes were considered inflammatory markers and have been used recently for patients with COVID-19^(8,10)^ We compared hemogram parameters (hemoglobin, leukocytes, lymphocytes, neutrophils, monocytes, and platelets), derived indices (NLR, LCRPR, NMR, LMR, and PLR), C-PCR, and creatinine levels between the two groups. The groups differed in the following: hemoglobin levels on days 0 and 5, with Group B having a lower value (D0: 13.36±1.63 *versus* 12.7±1.74; D5:13.02±1.69 *versus* 12.08±2.1); lymphocyte count on days 0 and 5 was higher in Group B, but in the normal range; and platelet count on days 0 and 5 (also in the normal range). On day 5, Group B had higher monocyte and leukocyte counts, with a mean leukocyte count above the normal range (9938.5±4429.3 *versus* 11942.9±5605.9, p=0.042). On day 5, Group A showed a higher NLR (mean=11.6) and PLR (mean=366.2). Hemoglobin, LCRPR, NLR, and CRP levels on day 0 did not affect the number of days of hospitalization, Mechanical ventilation (MV), or ICU stay (Tables 2 to 4).

**Table 2:** Cox proportional hazards regression model among patients with COVID-19 with days of intensive care stay as a dependent variable

Variable	HR	95%CI		p value
		Lower	Upper	
Group B	1.003	0.519	1.939	0.992
Dialysis (No)	14.493	4.405	47.619	<0.001*
WHO D0	0.789	0.625	0.997	0.047*
CPEVNT (x100)	0.985	0.953	1.018	0.366
Transfused volume (x100)	0.926	0.767	1.118	0.423
Hb D0	1.042	0.928	1.170	0.488
LCRPR D0	1.002	1.000	1.003	0.066
NLR D0	1.001	0.991	1.012	0.816
C-reactive protein D0	0.999	0.996	1.002	0.381

* p<0.05.

Cox Model. WHO D0: Word Health Organization ordinal scale on the day of transfusion; CPEVNT: cytopathic effect-based virus neutralization test; Hb D0: hemoglobin on the day of transfusion; L-CRP-R D0: lymphocyte-to-C-reactive protein ratio on the day of transfusion; NLR D0: neutrophil-to-lymphocyte ratio on the day of transfusion.

**Table 3:** Cox proportional hazards regression model among patients with COVID-19 with days of hospitalization as a dependent variable

Variable	HR	95%CI		p value
		Lower	Upper	
Group B	1.769	0.882	3.549	0.108
Dialysis (No)	18.519	4.425	76.923	<0.001*
WHO D0	0.661	0.521	0.839	0.001*
CPEVNT (x100)	0.990	0.959	1.022	0.540
Transfused volume (x100)	0.917	0.755	1.115	0.385
Hb D0	1.041	0.919	1.179	0.527
LCRPR D0	1.001	1.000	1.003	0.116
NLR D0	1.000	0.992	1.009	0.959
C-reactive-protein D0	1.000	0.997	1.003	0.879

* p<0.05.

Cox Model. WHO D0: Word Health Organization ordinal scale on the day of transfusion; CPEVNT: cytopathic effect-based virus neutralization test; Hb D0: hemoglobin on the day of transfusion; L-CRP-R D0: lymphocyte-to-C-reactive protein ratio on the day of transfusion; NLR D0: neutrophil-to-lymphocyte ratio on the day of transfusion.

**Table 4:** Cox proportional hazards regression model among patients with COVID-19 with days of mechanical ventilation use as a dependent variable

Variable	HR	95%CI		p value
		Lower	Upper	
Group B	1.161	0.601	2.241	0.657
Dialysis (No)	6.897	2.674	17.544	<0.001*
WHO D0	0.793	0.620	1.015	0.065
CPEVNT (x100)	0.988	0.957	1.020	0.464
Transfused volume (x100)	0.987	0.818	1.191	0.891
Hb D0	1.037	0.925	1.162	0.534
LCRPR D0	1.000	0.999	1.002	0.703
NLR D0	0.996	0.986	1.007	0.492
C-reactive-protein D0	0.999	0.996	1.002	0.517

* p<0.05.

Cox Model. WHO D0: Word Health Organization ordinal scale on the day of transfusion; CPEVNT: cytopathic effect-based virus neutralization test; Hb D0: hemoglobin on the day of transfusion; L-CRP-R D0: lymphocyte-to-C-reactive protein ratio on the day of transfusion; NLR D0: neutrophil-to-lymphocyte ratio on the day of transfusion.

To determine whether there was an improvement from day 0 to day 5, we analyzed the changes in the WHO ordinal scale. Group B (ΔWHO=-12.12, SD: 33.65) showed better clinical improvement than Group A (ΔWHO=13.99, SD: 30.88).

The global mortality rate was 12.1%, which did not differ significantly between the groups. The groups differed in the length of ICU stay (Group A=6.2, SD:9.8 *versus* Group B=14.6, SD:15.6) and the length of mechanical ventilation use (Group A=2.7, SD:5.65 vs. Group B=7.64, SD:13.23). The indications for dialysis did not significantly differ. To identify the factors that could influence outcomes, we used a Cox proportional hazards regression model, with the length ICU stay, hospitalization, and mechanical ventilation use as dependent variables (Tables 2 to 4). Using this model, we did not find that the CCP intervention affected the different outcomes in these groups. Dialysis negatively affected the length of ICU stay, hospitalization, and mechanical ventilation use. The WHO scale on day 0 also affected the results, reducing the probability of discharge from ICU by 21.1% and the probability of hospital discharge by 33.9% for each point higher on the scale. Additionally, the risk of mechanical ventilation discontinuation reduced by 20.7% for each higher point on the scale.

## DISCUSSION

This was a retrospective study with two groups of patients who received CCP: Group A, in which patients who received compassionate CCP (median volume 269 [150-435]; median nAbs 160 [80-2560], from January to May 2021, n= 94); and Group B, which included patients from a previous clinical study (median volume 600 [300-600]; median nAbs 320 [20-1810], from March to October 2020, n=47). Most participants were men, with a mean age >60 years and had comorbidities, which increase the risk of hospitalization, and ICU admission.^(14)^ Group B differed from Group A in that it had a higher percentage of patients in the ICU on the day of transfusion and a higher WHO ordinal scale score on day 0, suggesting greater disease severity.

Inflammation, including the use of CCP, is a problem in the treatment of COVID-19.^(7)^ The hemoglobin value differed between the groups on days 0 and 5. Despite the median being in the normal range, the minimum value in the total group was 7.9 mg/dL on day 0 and 7.6 mg/dL on day 5, possibly because of the anemic hypoxia, as was already described in COVID-19 infection.^(15)^ The median value of total lymphocytes was 805 cells/μL on day 0 and 1069 cells/μL on day 5. Lymphopenia is believed to indicate a defective response to the virus and is associated with mortality and ICU admission.^(8)^ The median NLR was >11, a value predictive of acute respiratory distress syndrome^(16)^ and severe diseases.^(17)^ Thrombocytopenia can be detected in patients with COVID-19;^(18)^ however, our results for both groups were within the normal range.

The median of the CRP (95.1mg/L) on day 0 was approximately 19 times the normal range, a value lower than that reported in a previous study^(12)^ but is predictive of mortality.^(19)^ In the same manner, the mean NMR was 22.9 (SD: 24.7), which is predictive of in-hospital mortality in severely ill patients with COVID-19.^(20)^

The percentage of mean variation in the WHO ordinal scale was negative in Group B, suggesting that patients in this group, who had a longer duration of symptoms (Table 1), had better clinical improvement. Despite these differences, Group B patients presented with a higher mean number of days in the ICU and on mechanical ventilation. However, although CCP was well tolerated, it did not show any influence on the outcomes, even with different volumes, nAb titers, and times from admission to transfusion. This is in agreement with the results of a previous study that found that convalescent plasma did not reduce the risk of intubation or death after 30 days in hospitalized patients with COVID-19.^(21)^ Additionally, a recent Cochrane review found that CCP did not reduce all-cause mortality up to day 28, had little to no impact on the need for invasive mechanical ventilation or on mortality, and did not affect whether the participants were discharged from the hospital.^(22)^

As shown in tables 2 to 4, dialysis influenced the length of hospitalization, ICU stay, and mechanical ventilation use. Damage to the renal system, the cause of kidney failure, can be caused by viral tropism, and the incidence of acute kidney injury in patients with COVID-19 could be as high as 25%,^(23)^ reaching 45% in ICU patients and affecting mortality.^(23,24)^ Acute renal injury is also associated with adverse outcomes in general ICU patients, such as increased length of stay and short- and long-term mortality.^(25,26)^ A higher WHO ordinal scale score on day 0 negatively affected the number of hospitalization and ICU discharge days and reduced the risk of mechanical ventilation discontinuation. To the best of our knowledge, this information is novel.

This study contributes to the current understanding of COVID-19. Similar to that reported in previous studies, CCP indeed has a safety profile similar to that of standard plasma, and can be implemented at the onset of future infectious disease outbreaks.^(6)^ Moreover, our patients had several severity markers that could impair their response to CCP treatment.^(7)^ Corticosteroids, which are used in most patients, can interfere with antibody function and affect the results.^(6)^

Nonetheless, this study had potential bias. This was a retrospective study with a limited number of patients and without a Control Group. Clinically, neither group was comparable, suggesting a selection bias. The mean number of days between symptom onset and transfusion was higher than five, and current data show that CCP is more effective when used early after symptom onset,^(5)^ partly justifying the lack of clinical response in this study. In addition, all patients were under clinical support and received other types of treatment, such as heparin, antiviral agents, or antibiotics, during hospitalization.

## CONCLUSION

Convalescent plasma did not affect the outcomes of patients with severe COVID-19 when comparing two different cohorts that were transfused with different volumes and titers of nAbs. Despite improvements in laboratory parameters, the clinical outcomes did not change. Dialysis negatively affected the length of intensive care unit stay, hospitalization, and mechanical ventilation use. Each higher point on the day 0 WHO scale reduced the probability of hospital and intensive care unit discharge and the risk of mechanical ventilation discontinuation. Based on this, dialysis and the assessed clinical severity represented by the WHO scale score on day 0 influenced the outcomes, but not convalescent plasma transfusion.
